# Exploring the dynamics of mobile app addiction: the interplay of communication, affective factors, flow, perceived enjoyment, and habit

**DOI:** 10.1186/s40359-023-01440-8

**Published:** 2023-11-20

**Authors:** Hyeon Jo, Eun-Mi Baek

**Affiliations:** 1 HJ Institute of Technology and Management, 71 Jungdong-ro 39, Bucheon-si, Gyeonggi-do 14721 Republic of Korea; 2 Department of Preventive Medicine, College of Medicine, Catholic University of Korea, 222 Banpo-daero, Seocho-gu, Seoul, 06591 Republic of Korea

**Keywords:** Mobile app, Addiction, Communication, Affect, Flow, Perceived enjoyment, Habit

## Abstract

The rapid proliferation of mobile apps and their increasing usage have led to growing concerns about potential addiction among users. Previous research has identified several factors that contribute to addiction, including flow, perceived enjoyment, and habit. However, the underlying mechanisms and the role of affective factors remain unclear. This study aims to elucidate the key factors that affect mobile app addiction by proposing a theoretical framework incorporating communication, affective factors, flow, perceived enjoyment, and habit. Data were collected from 320 mobile app users through a questionnaire survey. The research employed partial least squares structural equation modeling (PLS-SEM) to analyze the data and test the proposed relationships. The analysis indicates that communication is significantly associated with perceived enjoyment but does not directly affect flow and habit. The research unveils that positive affect significantly influences both flow and perceived enjoyment, but does not influence habit. Negative affect was found to have no significant effect on flow, perceived enjoyment, and habit. The results demonstrate that flow, perceived enjoyment, and habit are significantly related to addiction. The findings reveal that perceived enjoyment has a substantial impact on both flow and habit. These findings offer valuable guidelines for future research and practical implications for developers and policymakers in addressing the challenges associated with mobile app addiction.

**Supplementary Information:**

The online version contains supplementary material available at 10.1186/s40359-023-01440-8.

## Introduction

With the rise of technology, mobile applications have become an integral part of daily life, serving myriad purposes ranging from entertainment to productivity. These applications have permeated every facet of our existence, changing the way we communicate, work, play, and even perceive the world around us. As of 2018, the global mobile application market was valued at $106.27 billion and is projected to reach $407.31 billion by 2026, growing at a CAGR of 18.4% from 2019 to 2026 [1]. The Apple app market alone has registered 1.96 million apps [2]. In this fragmented market, mobile game apps constitute the highest percentage, followed by music entertainment, and social networking service (SNS) apps. On average, users worldwide spend 4.2 h a day using mobile apps, with 49% of users opening an app at least 11 times a day and 70% of all US digital media time spent on mobile apps [2]. The rapid increase in mobile usage has sparked both interest and concern over potential addiction.

Mobile applications are meticulously engineered to capture user attention and foster engagement [3]. Features like real-time notifications, endless scrolling, and gamification elements are quintessential in ensuring sustained user interaction [4]. Renowned platforms such as Facebook, Instagram, and mobile games like Candy Crush have been discerned to instigate addictive behaviors [5, 6]. For instance, the incessant checking of social media apps or the relentless pursuit of advancing game levels transpires despite the apparent adverse repercussions on users’ sleep, productivity, or even mental health [7]. The propensity for these behaviors underpins the ubiquitous nature of mobile app addiction. Owing to the omnipresence of mobile devices, the line between moderate and excessive use has become increasingly blurred, thereby escalating the necessity to delve into the factors contributing to such addiction. The ubiquitous nature of mobile app addiction is underscored by emerging research, which delineates the cognitive and behavioral tendencies driving this phenomenon [8, 9]. As the ramifications of mobile app addiction seep into various facets of daily life, the exigency of investigating the underlying factors and promoting healthier digital consumption patterns is accentuated.

Communication, particularly on mobile platforms, has been a transformative experience. It’s no longer merely about exchanging information but also about validation, status updates, and even maintaining one’s online persona. These apps, through likes, comments, and shares, offer instant gratification, potentially fueling addictive tendencies [10]. The act of constantly checking for messages or updates, even in the absence of notifications, is a clear indication of an underlying addiction to communication facilitated by mobile applications. As well, mobile app addiction necessitates a multi-faceted approach, where examining the role of emotional states like positive and negative affect becomes crucial. These affective states are pivotal as they significantly influence user interaction and engagement with mobile apps [11]. Positive affect may foster a conducive environment for addiction to burgeon. When users derive pleasure, satisfaction, or a sense of accomplishment from interacting with mobile apps, they are likely to use them more frequently and for extended durations [12]. On the contrary, negative affect could engender a scenario where individuals resort to mobile apps as a coping mechanism to mitigate stress, anxiety, or other unfavorable emotional states, which is a hallmark of escapist behavior often seen in addiction [13]. Analyzing positive and negative affect independently is imperative to delineate the unique pathways through which each affective state contributes to mobile app addiction. For instance, positive affect might be more associated with gratification-seeking behavior, while negative affect could be linked with avoidance or escapist behavior. This distinction, illuminated in several research studies [14], assists in a more nuanced understanding and intervention formulation to curb mobile app addiction.

Perceived enjoyment, signifying the intrinsic pleasure derived from utilizing an app, often acts as a pivotal driver enticing users to continually engage with it [15]. The allure of an enjoyable user interface can elicit a desire to revisit the app, potentially fostering a habitual or even addictive behavior [16]. The enjoyable elements, ranging from aesthetic appeal to interactive features, enhance the user experience, thereby cultivating a sense of gratification which can morph into a relentless pursuit for that gratification over time [17]. Thus, a deepened understanding of how perceived enjoyment intertwines with addiction can shed light on developing balanced app designs that ensure user engagement while mitigating addictive behaviors.

Flow often causes users to lose awareness of time, possibly resulting in excessive app usage [18]. Recent studies present a more detailed understanding of flow, characterized by deep immersion and engagement, indicating that its impact can occasionally be adverse. For example, Barberis et al. [19] explain that certain psychological needs may foster harmonious passion and consequently, a flow experience, while others might instigate an obsessive passion, leading to problematic behaviors. In a study on big wave surfers, Partington et al. [20] discovered that while flow led to positive outcomes like mood enhancement and performance improvement, it also correlated with a dependency on the activity. This dependency manifested to the extent that some surfers faced challenges functioning ‘normally’ within society due to their surfing engagement. This highlights the essential notion that while flow can enhance performance and engagement, it may also foster behavioral addiction, particularly as individuals continually chase the euphoric sensations associated with the flow experience. The captivating euphoria, especially when skills align with challenging tasks, can entice individuals to incessantly seek such experiences, leading to a potential obsession with the activity, as demonstrated in the surfers’ scenario.

Habit represents the routine engagement with mobile apps, a behavior often occurring without deliberate thought, thereby reinforcing addictive patterns [21]. This habitual interaction gradually becomes a fixed part of an individual’s daily routine, reducing the necessity for conscious decision-making regarding app usage [22]. The inherent automaticity in habit formation is significant, as it can transcend the initial voluntary interaction, propelling a cycle of compulsive usage over time [23]. With each repetitive engagement, the habit strength amplifies, potentially heightening the inclination towards addiction [24]. This continuous cycle accentuates the importance of delving into habit within the context of mobile app addiction. Unveiling the complex interplay between habit formation and addictive behavior may offer pivotal insights, aiding in the crafting of interventions to mitigate maladaptive app usage. Such intricate liaison positions habit as an indispensable construct necessitating thorough examination in the extensive narrative surrounding mobile app addiction, thus enriching understanding of the multifarious nature of user interaction with mobile apps. Given the above, the research questions this paper aims to address are:

How do communication and affect, while engaging with mobile applications, contribute to the manifestation of addictive behaviors?

What roles are played by perceived enjoyment, flow, and habit in nurturing addiction towards mobile app usage?

This research differentiates itself from prior studies in several ways, offering new contributions to the field. First, this study comprehensively examines addiction among mobile app users. Previous studies have examined addiction by dividing the types of apps and focusing mainly on addiction to certain types such as games [25, 26], commercial apps [27, 28], messengers [29–31], and SNS [32–35]. Recently, with the rapid spread of OTT services, instances of addictive video consumption through apps have been reported [36]. This study acknowledges that app users frequently switch between numerous apps. Users can become immersed in games, deeply engaged in messenger apps, or spend extended periods on social networks. This research offers new insights by examining addictive behavior for a broader range of apps, rather than focusing on a single type of app. Second, this research introduces both flow and habit simultaneously to explain addiction. Flow usually enhances performance [37] or encourages desirable behaviors [38]. Habits, on the other hand, often undermine work efficiency by promoting unconscious behavior [18]. This paper presents a balanced interpretation by reflecting on both positive and negative factors. Third, this study incorporates communication as an exogenous variable. Many mobile apps primarily serve communicative functions. Users communicate with other players in-game apps [39] or chat through messenger apps [40, 41]. They also engage with others through commenting functions on social network posts or expressing opinions on OTT online bulletin boards. Given that many apps include social functions, communication may play a critical role in mobile app addiction. Lastly, this study considers the emotional factors of app users. Previous studies on mobile app addiction did not delve deeply into the role of affect. Users often experience emotional changes through social comparison [42, 43]. Therefore, human affect could be an essential factor in the formation of addiction. The results of this study contribute to academia by illuminating the effects of emotions on addiction.

The structure of this study unfolds as follows: Sect. 2 details the background of the study. Section 3 describes the research model and each hypothesis. Section 4 presents the empirical methodology. Section 5 covers the research results. Section 6 discusses the results. Finally, Section 7 outlines the study’s implications and limitations.

## Theoretical background

The increasing prevalence of mobile applications has raised concerns about the potentially addictive nature of these technologies. Addiction to mobile applications refers to a state of compulsive use, characterized by an inability to control or reduce usage, negative consequences, and a preoccupation with the application [44, 45]. This paper explores the theoretical basis for adopting the antecedents of addiction to mobile applications.

### Flow theory

Flow, a concept introduced by Csikszentmihalyi [46], describes a unique cognitive state where individuals find themselves completely engrossed in an activity to the point that they lose sense of time and are detached from external distractions. This phenomenon occurs when there’s a harmonious balance between one’s skills and the challenges posed by the activity. Distinctively characterized by heightened concentration, clear goals, immediate feedback, and a sense of personal control over the situation, flow provides intrinsic rewards, enhancing enjoyment and satisfaction derived from the task [46].

Mobile applications, with their interactive interfaces and often instant gratification mechanisms, are well-primed to induce flow experiences [47]. By delivering an equilibrium of challenges tailored to user skill levels, these apps foster optimal conditions for flow, augmenting user engagement and retention [48].

However, the link between flow and addictive behaviors is complex. While flow provides enriching experiences, the intense immersion and emotional highs associated with it can lead to over-reliance and overuse of mobile applications. Several studies have documented how the positive emotions resulting from flow can heighten the risk of addiction. For instance, the profound immersion in flow experiences can mask the potentially detrimental consequences of excessive app use, making users less aware of the time spent or neglect of other responsibilities [45]. This aspect is particularly concerning as, over time, the pursuit of flow can transition from a healthy engagement to compulsive usage patterns, reflective of addiction [49].

Furthermore, flow’s intricate relationship with addictive behaviors has been examined in multiple contexts, beyond mobile apps. Research by Mehmet [50] and Salehan and Negahban [51] highlighted how online gamers, when experiencing flow, showed increased tendencies for prolonged gaming sessions, suggesting potential risks for gaming addiction. Similarly, Sun et al. [52] found that flow experiences in online shopping platforms could lead to impulsive buying behaviors. Trevino and Webster [53] discussed how online environments, specifically due to their interactive nature, can easily induce flow states, leading to prolonged durations of usage. While flow can enhance the richness of online experiences, there’s a growing concern about its association with excessive online behaviors. Wan and Chiou [54] argued that users, especially in online gaming environments, can get so captivated in the flow that they lose track of time, fostering addictive tendencies. This deep immersion, as indicated by Faiola et al. [55], might promote compulsive behavior in online platforms, making the line between healthy and excessive engagement increasingly thin.

Overall, these factors support the idea that flow acts as a precursor to addiction in the context of mobile applications, as users become fully engaged, motivated, and immersed in the experience, potentially leading to addictive patterns of use.

### Habit theory

Habit refers to a behavioral pattern that becomes automatic and repetitive through reinforcement [56]. According to habit theory, behaviors that are consistently repeated can become ingrained as automatic habits through reinforcement [56].

Mobile applications often employ cues, such as notifications, which trigger a response from users, leading to subsequent reinforcement, such as social rewards [23]. This cue-response-reinforcement loop can contribute to the formation of habits and, consequently, addiction. The accessibility of mobile applications in various contexts further facilitates the development of consistent usage patterns, allowing habits to form more easily [57]. Researchers have validated a significant association between habit and technology addiction [9, 15, 58]. As habits solidify, the associated behaviors become automatic and less reliant on conscious intent [56]. This automaticity may make it challenging for individuals to recognize and exert control over their addictive behaviors [59].

Through consistent reinforcement and contextual consistency, habitual behaviors are formed, making it difficult for individuals to consciously regulate their addictive tendencies.

### Perceived enjoyment

Perceived enjoyment, which refers to the level of pleasure or fun experienced during mobile application use [60], has been identified as a significant factor influencing addiction to mobile applications. Numerous factors support the idea that perceived enjoyment serves as an antecedent of addiction.

Perceived enjoyment is closely linked to the concept of flow, which represents a state of complete absorption and intrinsic motivation in an activity [61]. Several factors support the idea that perceived enjoyment serves as an antecedent of flow [62, 63]. Individuals are more likely to experience flow when they perceive an activity as enjoyable [61]. Mobile applications that provide enjoyable experiences can create an optimal balance between challenge and skill, facilitating full absorption and the experience of flow.

Perceived enjoyment can also contribute to the formation of habits related to mobile application use. Enjoyable experiences can act as rewards that reinforce the behavior of using a mobile application, leading to habit formation [23]. As individuals repeatedly experience enjoyment while using the application, the habit becomes stronger. With continued use, the behaviors associated with enjoyable mobile applications can become more automatic and less reliant on conscious intention [56, 64, 65]. This automaticity can facilitate the formation of habits, as the behaviors become ingrained in individuals’ daily routines.

Perceived enjoyment, often rooted in intrinsic motivation, has gained attention as a pivotal construct when evaluating user experiences online [66]. It’s posited that when users derive pleasure and gratification from an activity, their likelihood of continuous engagement increases [67]. However, this repeated engagement, propelled by heightened enjoyment, can sometimes cascade into compulsive behaviors and potential addiction. Several studies have demonstrated that while enjoyment can foster initial attraction to digital platforms, it’s also intricately linked to the spirals of addiction [15, 68, 69]. The allure of consistent positive feedback and pleasurable experiences inadvertently ropes individuals into cyclic patterns of excessive usage, highlighting the double-edged sword of perceived enjoyment.

In summary, perceived enjoyment plays a crucial role in addiction to mobile applications. It can enhance intrinsic motivation, contribute to the experience of flow, and facilitate habit formation. By understanding the factors that influence perceived enjoyment, researchers and practitioners can gain insights into the mechanisms underlying addictive behavior and develop strategies to promote healthy mobile application use.

### Communication

The role of communication in shaping addiction to mobile applications among university students is examined in this paper. Communication, which involves exchanging information, ideas, and emotions through various channels, plays a significant role in mobile application use [70]. Mobile applications provide platforms for social interaction and communication, which are essential aspects of university students’ lives [71]. The ease of communication facilitated by these applications can contribute to addiction as students become dependent on them to maintain social connections [72]. Additionally, mobile applications play a role in the formation and maintenance of social identity, allowing students to communicate with like-minded individuals, join communities, and share experiences [73]. The need to uphold and enhance social identity can contribute to addiction as students rely on mobile applications for this purpose [74].

Communication within mobile applications can facilitate flow experiences by providing a sense of social presence, and the feeling of being connected with others in a virtual environment [75]. This social presence enhances immersion and the likelihood of experiencing flow, which in turn contributes to addiction [61, 76]. Perceived enjoyment can be enhanced through communication features in mobile applications, as users derive pleasure from interacting with others [60, 77–79]. This increased enjoyment can contribute to addiction by strengthening the intrinsic motivation to use the application [80]. The repetitive nature of communication activities within mobile applications can further contribute to habit formation [56]. As students engage in frequent communication, the associated behaviors become more automatic and less reliant on conscious intent, potentially leading to habit formation [22] and addiction [59].

### Affect

The role of affect in shaping human behavior and experiences is widely recognized in psychological research [81–83]. Affect, encompassing positive and negative emotions, has a significant impact on various aspects of human functioning.

In the context of a flow, positive affect enhances the experience by promoting cognitive flexibility, creativity, and problem-solving abilities. Individuals experiencing positive emotions are better equipped to handle challenges and utilize their skills effectively, increasing their likelihood of experiencing flow [82, 84]. On the other hand, negative affect can hinder flow by impairing cognitive functioning and increasing anxiety, making it difficult for individuals to maintain focus and motivation [85].

Affect also plays a crucial role in shaping perceived enjoyment, which refers to the pleasure or fun individuals experience during an activity. Positive affect creates a positive atmosphere that enhances enjoyment and satisfaction, leading to increased intrinsic motivation. Individuals experiencing positive emotions are more likely to derive pleasure from activities and engage in them willingly. Conversely, negative affect creates a negative atmosphere that diminishes enjoyment and satisfaction, reducing intrinsic motivation and engagement in the activity. Furthermore, affect can influence the formation of habits, which are automated behavioral responses triggered by contextual cues. Positive affect reinforces behaviors through rewarding experiences, making individuals more likely to repeat those behaviors and strengthen the associated habit [86–88]. In contrast, negative affect can hinder habit formation by creating aversive experiences that discourage behavior repetition [89]. However, it’s important to note that negative affect can also contribute to habit formation when individuals engage in behaviors that provide temporary relief from negative emotions, potentially leading to maladaptive habits.

Overall, affect plays a crucial role in shaping human behavior and experiences, influencing the experience of flow, perceived enjoyment, and the formation of habits. Understanding the impact of affect can provide valuable insights into individuals’ motivation, engagement, and the development of behavioral patterns.

## Research Model

**Fig. 1:**
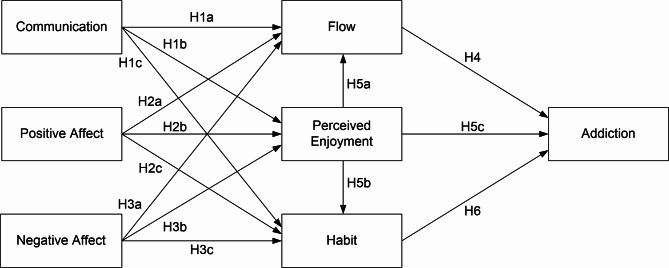
Research model

Figure 1 illustrates the research model delineating the determinants of mobile app addiction. It posits that communication, positive affect, and negative affect significantly influence the predictors of addiction. Moreover, this study investigates the roles of flow, perceived enjoyment, and habit in eliciting addiction.

### Communication

Communication, as the independent variable in this study, is conceptualized as the process by which individuals use mobile applications to share information, ideas, and feelings, thus facilitating social connectivity and interaction [90]. This involves using mobile apps to maintain contact with distant individuals, meet diverse people, and initiate conversations with new acquaintances. Numerous studies suggest a positive relationship between communication and flow [91, 92]. In the context of mobile app use, features that facilitate engaging, smooth, and immersive interactions can enhance the experience of flow [93]. For instance, the ability to have instant, seamless interactions with various people can increase users’ involvement, thereby contributing to an enhanced flow state [94]. Communication has also been found to have a significant relationship with perceived enjoyment [95]. Features that enhance communication can enrich the user’s experience, resulting in higher perceived enjoyment [77–79]. Enhanced connectivity and social interaction offered by mobile apps often lead to increased user enjoyment [96]. Communication can play a vital role in the formation of habits. Habits are automated behaviors that are triggered by consistent contextual cues [56]. Frequent and gratifying communication facilitated by mobile apps can create strong cues for habitual use. Continual social interactions can reinforce the habitual use of certain apps, embedding them in users’ daily routines [22]. Thus, this study suggests the following hypotheses.

H1a. Communication is positively correlated with flow.

H1b. Communication is positively correlated with perceived enjoyment.

H1c. Communication is positively correlated with habit.

### Positive affect

Positive affect is characterized as the extent to which an individual feels enthusiastic, active, and alert, encompassing a variety of pleasurable engagements with the environment [97]. Positive emotions can broaden an individual’s momentary thought-action repertoire, promoting exploratory behavior and open-minded thinking [83]. This expanded mindset can facilitate an individual’s engagement and absorption in an activity, leading to an increased experience of flow. Researchers have empirically proven that positive affect promotes the flow state [82, 84]. According to the Broaden-and-Build theory, positive emotions broaden individuals’ thought-action repertoires, building their enduring personal resources, including psychological well-being and enjoyment [83]. Enjoyment is associated with positive affect because enjoyable activities often induce positive emotions [98 1999]. Furthermore, positive emotions can enhance habit formation [86–88]. Positive affect tends to reinforce behavior, making it more likely to become habitual [99]. Additionally, habitual behaviors are likely to be carried out again when they are associated with positive feelings [100]. Thus, this study suggests the following hypotheses.

H2a. Positive affect is positively correlated with flow.

H2b. Positive affect is positively correlated with perceived enjoyment.

H2c. Positive affect is positively correlated with habit.

### Negative affect

Negative affect is defined as a general dimension of subjective distress and unpleasurable engagement that subsumes a variety of aversive mood states, including anger, contempt, disgust, guilt, fear, and nervousness [97 1988]. Flow, a state of complete absorption and enjoyment in an activity, is likely to be hampered by negative emotions, such as anxiety and stress [85]. Negative emotions can restrict an individual’s ability to fully immerse themselves in an activity, thus reducing the potential for experiencing flow [101]. Negative emotions, such as anxiety, stress, and sadness, can lower individuals’ satisfaction and enjoyment derived from an activity [102]. In the context of mobile app usage, negative affect may reduce the overall enjoyment perceived by users. Research has shown that negative emotions can inhibit habitual behavior, as these emotions often prompt individuals to reassess their actions and potentially alter their behaviors [89]. Therefore, persistent negative affect may deter the formation of habits in mobile app usage among university students. Hence, based on these arguments, the following is proposed in this study:

H3a. Negative affect is negatively correlated with flow.

H3b. Negative affect is negatively correlated with perceived enjoyment.

H3c. Negative affect is negatively correlated with habit.

### Flow

Flow, as defined, signifies a state of total immersion and concentrated energy that is fully engaged in the present activity [46]. It’s typified by intense concentration, the loss of self-consciousness, a sense of control, and the feeling that time is swiftly passing. Recent literature abounds with evidence that flow experience notably contributes to technology-related addictions, including mobile apps [47, 50–52]. As users enter a state of flow, they often lose track of time and neglect other tasks, potentially increasing their reliance on mobile apps [49]. Moreover, the flow state can induce compulsive usage of mobile apps, which can eventually evolve into addiction [103]. Thus, this study posits the following hypothesis.

H4. Flow is positively correlated with addiction.

### Perceived enjoyment

Perceived enjoyment, as defined, is the extent to which the activity of using a computer is perceived to be enjoyable apart from any performance consequences resulting from system use [60]. It’s seen as a significant intrinsic motivation for information system usage [60]. Perceived enjoyment can amplify the flow experience [62, 63]. Moreover, recent research indicates that perceived enjoyment is a vital predictor of technology addiction, which includes mobile apps, where users persist in engaging with the activity despite adverse consequences [52, 58, 104]. Additionally, evidence suggests that enjoyment can bolster habitual behaviors [64, 65]. Hence, this study forwards the following propositions.

H5a. Perceived enjoyment is positively correlated with flow.

H5b. Perceived enjoyment is positively correlated with habit.

H5c. Perceived enjoyment is positively correlated with addiction.

### Habit

Habit is characterized as the extent to which individuals tend to automatically perform behaviors due to learning [100]. Consistent research over recent years has demonstrated a significant relationship between habit and technology addiction, including mobile apps [9, 15, 58]. Studies show that as mobile app usage becomes more habitual, it is more likely to result in addictive behavior [23, 105, 106]. Habitual behavior can elicit an automatic response to cues related to app usage, thereby increasing the chances of addiction [107, 108]. Thus, this study puts forth the subsequent hypothesis.

H6. Habit is positively correlated with addiction.

## Methodology

### Instrument development

To ensure an accurate and reliable understanding of each construct, we utilized scales previously established in the literature. Each construct was measured using specific scales, as follows:

For the construct of communication, we adopted the scale developed by Abrahim et al. [109]. This scale comprises three items. Specifically, the items include CMU1, which states “I can get closer to people I don’t see often through mobile apps”, CMU2, “I can meet various people through mobile apps”, and CMU3, “Through mobile apps, I can have a conversation with people I meet for the first time.“ Respondents rated these statements on a Likert scale ranging from 1 (strongly disagree) to 5 (strongly agree). A higher score on this scale denotes stronger feelings of connectivity and communication through the use of mobile apps.

In measuring positive affect, we utilized the scale from Watson et al. [97]. This scale consists of three items. They are POA1: “I am passionate while using mobile apps”, POA2: “I am proud while using mobile apps”, and POA3: “I get inspiration while using mobile apps.“ Ratings were done on a Likert scale from 1 (strongly disagree) to 5 (strongly agree). notably, a higher score in this construct represents an elevated level of positive emotions in relation to mobile app usage.

To gauge negative affect, we again turned to the scale by Watson et al. [97]. With three items, exemplified by NEA1, “I suffer while using mobile apps”, NEA2, “I get angry while using mobile apps”, and NEA3, “I feel annoyed while using mobile apps”, participants expressed their agreement on a Likert scale from 1 (strongly disagree) to 5 (strongly agree). It’s pertinent to note that higher scores here indicate an increased sentiment of distress or negative emotions tied to the usage of mobile apps.

For flow, the scale designed by Jo [110] was chosen. This scale contains three items. Among them, FLW1 states “While using mobile apps, my attention was focused solely on them”, FLW2 expresses “I was completely focused while using mobile apps”, and FLW3 articulates “I was deeply immersed in mobile apps while using them.“ Participants’ responses were gathered on a Likert scale that spanned from 1 (strongly disagree) to 5 (strongly Agree). In this construct, higher scores signify a more profound state of flow and immersion during the usage of mobile apps.

Perceived enjoyment was measured using the scale formulated by Davis et al. [60]. It has three items, exemplified by PEN1, “It is fun to use mobile apps”, PEN2, “Using mobile apps is interesting”, and PEN3, “Using mobile apps gives me pleasure.“ Again, participants voiced their agreement on a Likert scale from 1 (strongly disagree) to 5 (strongly agree). A notable point is that higher scores in this domain mirror an enhanced sense of enjoyment derived from the apps.

For the construct of Habit, the scale crafted by LaRose and Eastin [22] was the choice. It incorporates three items. They include HAB1, which claims “I use mobile apps to kill time”, HAB2, “I habitually use mobile apps whenever I have spare time”, and HAB3, “I use mobile apps to relieve boredom.“ Ratings took place on a Likert scale ranging between 1 (strongly disagree) and 5 (strongly agree). Here, it’s crucial to observe that higher scores suggest a more embedded habit of mobile app utilization.

Lastly, to assess addiction, the scale by Karadağ et al. [111] was adopted. This scale encompasses two items: ADD1, “I was immersed in mobile apps and experienced a decrease in conversations when meeting people” and ADD2, “As I used mobile apps, the emotional and affectionate emotions of the past decreased.“ Participants provided their responses on a Likert scale from 1 (strongly disagree) to 5 (strongly agree). In this domain, elevated scores pinpoint stronger indications or tendencies of addiction to mobile apps.

To ensure the content validity of our survey instrument, the questionnaire was critically reviewed and refined by two researchers specializing in the Information Systems field before being disseminated. Detailed information on each construct’s items, along with their mean and source, is presented in Table 1.

**Table 1:** List of model constructs and items

Construct	Items	Mean	Source
Communication	CMU1	I can get closer to people I don’t see often through mobile apps.	[109]
	CMU2	I can meet various people through mobile apps.	
	CMU3	Through mobile apps, I can have a conversation with people I meet for the first time.	
Positive Affect	POA1	I am passionate while using mobile apps.	[97]
	POA2	I am proud while using mobile apps.	
	POA3	I get inspiration while using mobile apps.	
Negative Affect	NEA1	I suffer while using mobile apps.	[97]
	NEA2	I get angry while using mobile apps.	
	NEA3	I feel annoyed while using mobile apps.	
Flow	FLW1	While using mobile apps, my attention was focused solely on them.	[110]
	FLW2	I was completely focused while using mobile apps.	
	FLW3	I was deeply immersed in mobile apps while using them.	
Perceived Enjoyment	PEN1	It is fun to use mobile apps.	[60]
	PEN2	Using mobile apps is interesting.	
	PEN3	Using mobile apps gives me pleasure.	
Habit	HAB1	I use mobile apps to kill time.	[22]
	HAB2	I habitually use mobile apps whenever I have spare time.	
	HAB3	I use mobile apps to relieve boredom.	
Addiction	ADD1	I was immersed in mobile apps and experienced a decrease in conversations when meeting people.	[111]
	ADD2	As I used mobile apps, the emotional and affectionate emotions of the past decreased.	

### Data collection

To investigate the factors influencing mobile app addiction, this study utilized a survey-based research methodology targeting university students. University students were chosen as the target population due to their representation of heavy mobile app users and previous reports highlighting their susceptibility to smartphone addiction. The survey underwent pre-testing with a small group of frequent mobile app users (n = 30) to ensure clarity and relevance. Based on feedback, minor modifications were made to enhance comprehension and eliminate potential ambiguity in the survey questions. Following a successful pilot test, the final survey was distributed offline in university classes. Participation was voluntary, and participants were provided with information about the study’s objective and the confidentiality of their responses.

For our study, we primarily focused on users who have previously experienced symptoms of addictive usage related to mobile apps. To identify such participants, we initially posed questions based on the addiction metrics adopted in our paper. If respondents indicated having had such experiences in the past, we included them in the study. Specifically, in the preliminary part of our questionnaire for screening purposes, we inquired with two statements: “I have experienced that when I was immersed in mobile apps, I engaged less in conversations when meeting people.“ and “I have experience that when as I used mobile apps, my emotional and affectionate feelings from the past diminished.“ Participants who responded affirmatively to at least one of these statements were included in our study.

The survey was distributed to a total of 400 students, resulting in 334 responses collected over one month. After removing incomplete and inconsistent responses, a total of 320 responses were considered for the analysis of hypothesized paths. The sample size criterion was determined using power analysis for structural equation modeling (SEM), following the guidelines of Hair et al. [112] and Munerah et al. [113]. G*power analysis was employed to calculate the minimum required sample size. With input values of a 0.15 effect size, 0.05 probability level, 95% desired statistical power level, and three predictors, the required sample size was determined to be 119, indicating that this criterion was adequately met.

The final sample consisted of 152 male respondents and 168 female respondents. Table 2 provides demographic information regarding the age distribution of the participants, with the majority falling within the 18–35 age range, which aligns with the primary demographic of heavy mobile app users [114].

**Table 2:** Profile of respondents

Demographics	Item	Subjects (*N* = 320)	
		Frequency	Percentage
Gender	Male	152	47.5%
	Female	168	52.5%
Mobile app usage time per day (Average)	< 30 min	26	8.1%
	30 min − 1 h	53	16.6%
	1 − 3 h	130	40.6%
	> 3 h	111	34.7%

## Results

This study demonstrated the measurement model and the structural model by using the partial least squares structural equation modeling (PLS-SEM) through SmartPLS 3.3.5 [115]. PLS was chosen due to its robustness and less restriction on the distribution of data and sample size [116]. It has been figured out to be useful in the social sciences such as information systems, marketing, and service management [117, 118].

### Measurement model

This study assessed the reliability and validity of the measurement model. To evaluate reliability, this study examined Cronbach’s alpha and composite reliability (CR). If Cronbach’s alpha is greater than 0.6 [119] and CR is higher than 0.7 [120], the reliability is satisfied. As shown in Table 3, Cronbach’s alpha and CR values of all the constructs are well over the expected threshold.

This study explored convergent validity and discriminant validity to confirm the validity of the measurement model. Convergent validity was confirmed by investigating both the average variance extraction (AVE) and the factor loads of the items associated with each construct. AVE values ranged between 0.620 and 0.897 which are higher than the expected threshold of 0.5 [120]. Factor loadings ranged from 0.728 to 0.959 and are all statistically significant at the p = 0.001 levels, presenting a satisfactory level of convergent validity [121]. Discriminant validity was satisfied since the square root value of AVE for each construct was greater than any other corresponding rows and column entries [120]. Table 4 shows the analysis results of discriminant validity.

**Table 3:** Scale reliabilities

Construct	Items	Mean	St. Dev.	Factor Loading	Cronbach’s Alpha	CR	AVE
Communication	CMU1	3.581	1.003	0.770	0.690	0.829	0.620
	CMU2	3.463	1.024	0.858			
	CMU3	3.075	1.121	0.728			
Positive Affect	POA1	2.425	0.997	0.875	0.816	0.889	0.727
	POA2	2.013	0.859	0.846			
	POA3	2.250	0.949	0.837			
Negative Affect	NEA1	1.788	0.728	0.928	0.943	0.963	0.897
	NEA2	1.756	0.781	0.959			
	NEA3	1.800	0.857	0.954			
Flow	FLW1	2.831	0.957	0.920	0.916	0.947	0.856
	FLW2	2.881	0.983	0.932			
	FLW3	2.944	0.989	0.924			
Perceived Enjoyment	PEN1	3.531	0.806	0.933	0.922	0.950	0.865
	PEN2	3.625	0.789	0.922			
	PEN3	3.581	0.818	0.935			
Habit	HAB1	4.056	0.861	0.762	0.790	0.875	0.701
	HAB2	4.013	0.908	0.828			
	HAB3	4.156	0.738	0.915			
Addiction	ADD1	3.500	1.194	0.907	0.693	0.865	0.762
	ADD2	3.225	1.214	0.838			

**Table 4:** Correlation matrix and discriminant assessment

Construct	1	2	3	4	5	6	7
1. Communication	0.787						
2. Positive Affect	0.094	0.853					
3. Negative Affect	-0.273	0.346	0.947				
4. Flow	0.002	0.362	0.268	0.925			
5. Perceived Enjoyment	0.264	0.356	0.079	0.376	0.930		
6. Habit	0.172	0.143	0.123	0.305	0.383	0.838	
7. Addiction	0.054	0.167	0.169	0.384	0.073	0.351	0.873

Note: Diagonal values are the square root of AVE

### Structural model

**Fig. 2:**
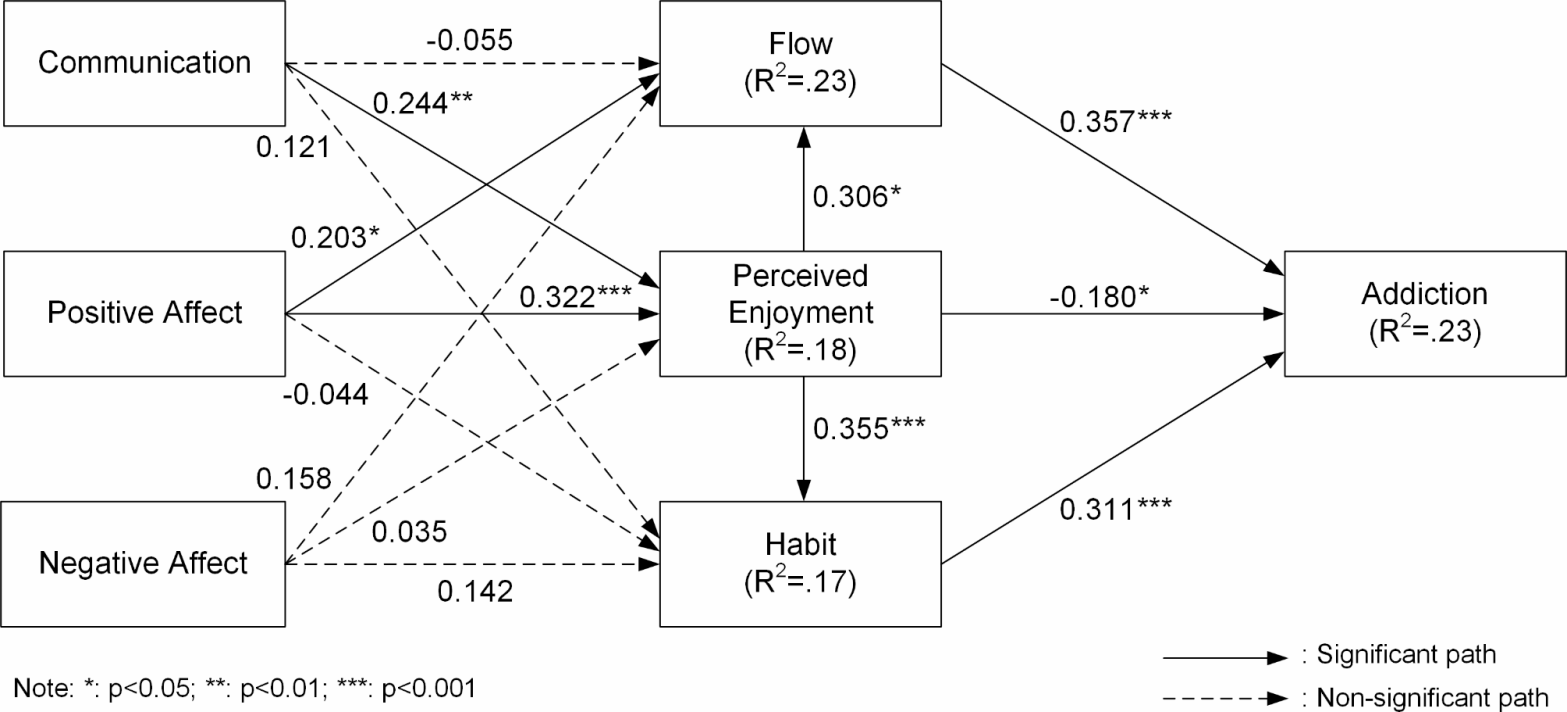
Results of structural modeling

The hypotheses in this study were evaluated utilizing the PLS-SEM technique provided by SmartPLS 3.3.5 for Windows. This research implemented a bootstrapping resampling method with 5,000 resamples. Out of the fourteen hypotheses presented in the research framework, eight received support. Figure 2 illustrates the results of analysis.

Table 5 summarizes the results of the hypothesis testing. The coefficient, *T*-value, and *P*-value are provided for each of the hypotheses tested. For the hypotheses examining the impact of communication on flow (H1a), perceived enjoyment (H1b), and habit (H1c), only the link between communication and perceived enjoyment was found to be statistically significant (p < 0.05). Similarly, while positive affect was found to significantly influence flow (H2a) and perceived enjoyment (H2b), it did not significantly affect habit (H2c). Negative affect did not have a significant influence on flow (H3a), perceived enjoyment (H3b), or habit (H3c). In terms of the hypotheses related to the impacts on addiction, flow was found to significantly influence addiction (H4), as did perceived enjoyment (H5c) and habit (H6). The effects of perceived enjoyment on flow (H5a) and habit (H5b) were also found to be statistically significant. Overall, the proposed model explained 23.3% of the variance in addiction.

**Table 5:** Summary of the Results

H	Cause	Effect	Coefficient	*T*-value	*P*-value	Hypothesis
H1a	Communication	Flow	-0.055	0.525	0.600	Not Supported
H1b	Communication	Perceived Enjoyment	0.244	2.932	0.003	Supported
H1c	Communication	Habit	0.121	1.317	0.188	Not Supported
H2a	Positive Affect	Flow	0.203	2.311	0.021	Supported
H2b	Positive Affect	Perceived Enjoyment	0.322	4.561	0.000	Supported
H2c	Positive Affect	Habit	-0.044	0.490	0.624	Not Supported
H3a	Negative Affect	Flow	0.158	1.613	0.107	Not Supported
H3b	Negative Affect	Perceived Enjoyment	0.035	0.392	0.695	Not Supported
H3c	Negative Affect	Habit	0.142	1.868	0.062	Not Supported
H4a	Flow	Addiction	0.357	4.305	0.000	Supported
H5a	Perceived Enjoyment	Flow	0.306	3.856	0.000	Supported
H5b	Perceived Enjoyment	Habit	0.355	3.801	0.000	Supported
H5c	Perceived Enjoyment	Addiction	-0.180	2.209	0.027	Supported
H6	Habit	Addiction	0.311	3.500	0.000	Supported

## Discussion

This study aimed to explore the factors influencing addiction among mobile app users, primarily through the lens of flow, perceived enjoyment, and habit. The results generated offer both insightful findings and unexpected contradictions concerning previous research.

The lack of support for H1a and H1c suggests that communication does not significantly influence flow and habit among university students using mobile apps, challenging previous studies that identified communication as a key driver for flow [93, 122] and habit [22]. This discrepancy might be attributed to differing communication patterns among contemporary university students, or to the possibility that their engagement with mobile apps is driven by factors beyond communication alone. In contrast, the hypothesis stating that communication positively influences perceived enjoyment (H1b) was supported, aligning with prior research suggesting that effective communication can enrich the user’s experience, thus increasing enjoyment [77–79]. This underscores the relevance of communication features in mobile apps, particularly those that enhance user experience and enjoyment.

Interestingly, while positive affect significantly influenced flow (H2a) and perceived enjoyment (H2b), consistent with prior studies [82, 84, 86–88], it did not significantly affect habit (H2c). This suggests that other factors might play a more crucial role in the formation of habitual behavior.

The results also indicated that negative affect did not significantly influence flow, perceived enjoyment, or habit (H3a, H3b, and H3c), contradicting previous research dealing with the association between negative affect and flow [101], perceived enjoyment [102], or habit [89]. This divergence suggests that the impact of negative emotions on app usage might be context-dependent or influenced by moderating variables not accounted for in this study. A potential explanation could be that mobile app use often serves as an escape from negative emotions [123, 124]. Thus, university students might use mobile apps as a coping mechanism when experiencing negative affect, leading to an increase, rather than a decrease, in flow, perceived enjoyment, and habit. Moreover, individual resilience or self-regulation capacities could have played a role [125, 126].

Flow (H4) and habit (H6) were found to positively influence addiction, aligning with previous studies [9, 15, 47, 50–52, 58]. Students who use mobile apps more immersively or habitually are thus more likely to develop an addiction.

Contrary to the initial hypothesis, perceived enjoyment appeared to negatively influence addiction among university students. This finding is somewhat counterintuitive, given prior research suggesting a positive relationship between perceived enjoyment and addictive behaviors in technology use [52, 58, 104]. The nuances of this relationship, however, are unpacked through the lens of harmonious passion, as delineated by [127]. Within this framework, profound enjoyment doesn’t inevitably spiral into obsessive engagement. Instead, when individuals derive genuine joy from an activity, they might engage in it in a more balanced and autonomous manner, thereby possibly reducing the inclination towards addiction. This perspective posits that genuine enjoyment fosters a holistic absorption, which contrasts with the compulsion typically associated with addiction. Furthermore, the role of individual differences, particularly self-control, cannot be understated. a study by Wang and Chu [128] highlights the duality of passion in online gaming, grounded in Vallerand et al. [127]’s framework. Their empirical results differentiate between harmonious passion, which usually doesn’t lead to addiction, and obsessive passion, which often results in addictive behaviors. Hence, it’s clear that only obsessive passion is associated with negative outcomes, such as addiction, within the realm of online gaming. The study by Stoeber et al. [129] explored the Dualistic Model of Passion in the context of online gaming, especially massively multiplayer online role-playing games (MMOs). Their findings reinforced the established understanding: harmonious passion for online gaming is associated with positive emotions during play, while obsessive passion predicts negative emotions both during play and when one is prevented from playing. In summary, our findings underscore the intricate dynamics between enjoyment and addiction, suggesting that genuine enjoyment, coupled with self-control, might act as protective factors against addictive behaviors.

Additionally, the positive effects of perceived enjoyment on flow and habit (H5b and H5c) further underscore the central role of enjoyment in mobile app usage. This may be because users experiencing a higher degree of perceived enjoyment are more likely to achieve a state of flow and, in turn, use mobile apps more habitually. These findings reiterate the critical role of flow, perceived enjoyment, and habit in understanding the mechanism of mobile app addiction among university students.

In conclusion, this study provides a fresh perspective on the dynamics of mobile app addiction among university students, highlighting the complex interplay of factors such as communication, positive and negative affect, flow, perceived enjoyment, and habit. The unexpected findings point to the need for future research to further explore these relationships, accounting for potential moderating variables and context-dependent nuances.

## Conclusion

### Theoretical contributions

This study makes a significant theoretical contribution by elucidating the relationships between communication, positive and negative affects, flow, perceived enjoyment, habit, and addiction in the context of mobile app usage among university students. Traditionally, research in this field has separately examined these variables, with limited consideration given to the intricate interplay between them. For instance, previous studies have established the influence of flow [93, 130], positive affect [84], and communication [77] on user enjoyment and habitual behavior. However, they have not concurrently analyzed these constructs in a comprehensive model. By integrating these factors into a single conceptual framework, this study not only confirms some of the findings from earlier works but also uncovers novel insights into the dynamics of mobile app usage and addiction.

A central contribution of this study lies in the unexpected finding that perceived enjoyment negatively affects addiction, contrary to the initial hypothesis and the results from previous studies [52, 58, 104]. This finding prompts a reconsideration of the role of enjoyment in addictive behaviors. Existing literature predominantly suggests a positive correlation between enjoyment and addiction, based on the assumption that higher levels of enjoyment lead to excessive usage and potential addictive behavior. The present study, however, suggests that individuals who experience higher levels of enjoyment might also possess better self-awareness and control over their usage, thereby reducing the likelihood of addiction. These results call for future research to explore potential moderating variables in the relationship between enjoyment and addiction, such as self-regulation or harmonious passion [127].

Another significant finding is the lack of a significant relationship between negative affect and flow, perceived enjoyment, or habit. Earlier studies have suggested a negative influence of negative affect on these factors, as adverse emotional states are often seen as detrimental to enjoyment and habit formation [89, 101, 102]. However, this study indicates that the influence of negative affect might be more nuanced and possibly context-dependent. In some circumstances, negative affect might not lead to decreased flow, perceived enjoyment, or habit. This discrepancy calls for a more nuanced understanding of the role of negative affect in mobile app usage and addiction, potentially considering moderating variables such as resilience [125] and self-regulation [126].

This study also enriches the theoretical understanding of the role of communication in perceived enjoyment and mobile app addiction. While communication has been widely recognized as an important factor in social media and gaming contexts [77, 78], its influence in the broader domain of mobile app usage has been understudied. The present study fills this gap by demonstrating that effective communication within mobile apps can increase user enjoyment and potentially contribute to habitual usage and addiction. Future studies could further explore the role of communication in different types of mobile apps, considering various communicative features and their impact on user experience.

Finally, by comprehensively integrating flow, perceived enjoyment, and habit, this study highlights the multi-faceted nature of mobile app addiction. While previous studies often adopted a piecemeal approach to these constructs [9, 15, 50], this study underscores their interrelationships and relative importance in the development of addiction. Specifically, this study found that flow and habit exert significant influence on addiction, underscoring the integral role of these factors in the path to addiction. While these findings corroborate previous research [9, 15, 47, 51, 52, 58], they also suggest the importance of assessing these constructs together. This holistic approach offers a more nuanced perspective on mobile app addiction, recognizing the complexity of factors influencing addictive behaviors. Furthermore, this research underscores the intricate dynamics between perceived enjoyment, flow, and habit, suggesting a strong interconnection between these constructs. This aligns with prior research indicating the significant influence of perceived enjoyment on flow and habit [131, 132]. However, by assessing these relationships within the larger model of mobile app addiction, this study offers a more comprehensive understanding of these dynamics. This finding implies that, while perceived enjoyment might mitigate addiction directly, it may also indirectly promote addiction by fostering flow and habitual behavior.

### Practical implications

This study’s practical implications are significant not only for stakeholders like app developers, educators, parents, and policy-makers but also for clinicians who aim to understand and address mobile app addiction in therapeutic contexts. The first practical implication pertains to the design of mobile apps. The fact that flow significantly contributes to mobile app addiction suggests that app developers need to balance the immersive experiences they create. While achieving a state of flow can result in a highly engaging user experience, it can also foster addictive behaviors [47, 50, 52]. Therefore, app developers might need to incorporate features that encourage conscious use and promote healthy digital habits. For example, they could consider integrating features that help users monitor their usage, set usage limits, or schedule breaks [133]. This could help users manage their engagement with the app and prevent the transition from immersive use to addictive behaviors. Further, clinicians should be aware of this risk when assessing patients’ digital habits, especially those who describe being deeply engaged or “in the zone” while using mobile apps. Interventions might focus on helping individuals recognize and manage instances of flow, ensuring it doesn’t contribute to excessive or unhealthy app usage.

Secondly, this study’s findings underline the importance of enhancing perceived enjoyment without fostering addiction. Given that perceived enjoyment was found to negatively influence addiction, service providers could focus on enhancing the enjoyment factor in their apps without necessarily increasing addictive tendencies [127]. They can achieve this by providing enjoyable and enriching content, improving user interface and interactivity, and offering personalized experiences that cater to individual preferences and interests [52]. Also, providers can maintain an open line of communication with their users to gather feedback and continuously improve the user experience, thereby increasing perceived enjoyment [77].

The third practical implication revolves around the positive impact of communication on perceived enjoyment. Service providers, especially those offering social networking services or multiplayer games, should prioritize enhancing their platforms’ communication features. This might involve improving chat functionalities, adding features that promote social interaction, and ensuring a safe and respectful communication environment [78, 79]. By doing so, service providers can increase user satisfaction, extend usage duration, and possibly promote more balanced and less addictive engagement with their platforms. In a clinical setting, it’s vital to assess the degree to which patients depend on mobile apps for their communication needs. Therapeutic strategies could target diversifying communication methods, promoting face-to-face interactions, and setting boundaries on app-based communication to mitigate addiction risks.

Moreover, the role of positive affect in promoting flow and perceived enjoyment suggests that creating a positive emotional environment is key in app design. Positive affect can be stimulated by incorporating elements of fun, humor, creativity, and achievements into the app’s features and content [82, 84]. By doing so, service providers could increase user engagement and satisfaction without necessarily fostering addictive behaviors. At the same time, they should be cautious about the potential risk of over-stimulation, which might, in turn, promote addictive usage patterns. Clinicians should probe into the emotional gratifications patients receive from certain activities, understanding that these positive emotions, while generally beneficial, can also be a slippery slope towards addiction. Treatment may involve helping patients find balance, ensuring they derive positive emotions from a range of activities and not solely from potentially addictive sources.

Lastly, the lack of impact of negative affect on flow perceived enjoyment, and habit suggests that app developers should focus more on fostering positive experiences rather than mitigating negative ones [89, 101]. However, this does not mean that negative experiences should be entirely overlooked. Service providers should continue striving to prevent or address issues that can lead to negative user experiences, such as cyberbullying, privacy violations, or technical issues, as they can potentially harm user satisfaction and loyalty [134].

In conclusion, the practical implications of this study are centered on promoting balanced and healthy mobile app usage. By considering these recommendations, various stakeholders can contribute to mitigating the growing issue of mobile app addiction.

### Limitation and further research

The limitations of this study and recommendations for further inquiry are as follows. A primary limitation pertains to the correlational nature of our findings. We emphasize that our results indicate associations rather than causations. Additionally, our sample size was fairly limited, potentially affecting the generalizability of our findings. Future studies with larger and more diverse samples are needed to validate our results. With respect to the study’s design, we did not account for the motivations of mobile app users, which can be pivotal in addiction. Users with hedonic motivations might display a higher susceptibility to addiction compared to those driven by utilitarian motivations. Furthermore, addiction-contributing factors could differ across these motivation-driven user groups. It would be beneficial for upcoming research to segment users based on their motivations and conduct multi-group analyses to gauge the repercussions on addiction. Another overlooked aspect was the demographic details of the participants. Given that demographic factors like age can influence addiction susceptibility – with adolescents potentially having diminished self-control compared to adults – future studies should incorporate these as control variables. Integrating demographics could enrich the academic depth of the findings. Conclusively, venturing into other potential influencers of mobile app addiction, like social stimuli, intrinsic motivations, or tech-related variables (e.g., app interface design), would be beneficial. A holistic theoretical framework will pave the way for a nuanced comprehension of this intricate behavior, which in turn will aid in devising potent interventions to curb and address mobile app addiction.

### Electronic supplementary material

Below is the link to the electronic supplementary material.

Supplementary Material 1

## Data Availability

The datasets used and/or analyzed during the current study are available from the corresponding author upon reasonable request.

## References

[1] Allied Market Research. Mobile application market statistics. 2019. https://www.alliedmarketresearch.com/mobile-application-market. Accessed 15 July 2022.

[2] Buildfire. Mobile app download statistics & usage statistics. 2022. https://buildfire.com/app-statistics/. Accessed 18 Jan 2022.

[3] Stocchi L (2022). Marketing research on Mobile apps: past, present and future. J Acad Mark Sci.

[4] Huang Z, Tian Z (2018). Analysis and design for Mobile Applications: a user experience Approach.

[5] Henzel V, Håkansson A (2021). Hooked on virtual social life. Problematic social media use and associations with mental distress and addictive disorders. PLoS ONE.

[6] Navyashree S, Vashisth S, Mishra W (2022). Game addiction and game design: a study based candy crush Saga players.

[7] Pellegrino A, Stasi A, Bhatiasevi V. Research trends in social media addiction and problematic social media use: a bibliometric analysis. Front Psychiatry. 2022;13:1017506. 10.3389/fpsyt.2022.1017506 PMC9707397 36458122

[8] Naslund JA (2020). Social Media and Mental Health: benefits, risks, and opportunities for Research and Practice. J Technol Behav Sci.

[9] Pera A. The psychology of addictive smartphone behavior in young adults: problematic use, social anxiety, and depressive stress. Front Psychiatry. 2020;11:573473. 10.3389/fpsyt.2020.573473 PMC7522217 33101087

[10] Marengo D (2021). Exploring the associations between Self-reported tendencies toward Smartphone Use Disorder and Objective recordings of Smartphone, Instant Messaging, and Social networking app usage: Correlational Study. J Med Internet Res.

[11] Jo H. Antecedents of continuance intention of social networking services (SNS): utilitarian, hedonic, and social contexts. Mob Inf Syst. 2022:7904124.

[12] Harrison R, Flood D, Duce D (2013). Usability of mobile applications: literature review and rationale for a new usability model. J Interact Sci.

[13] Kardefelt-Winther D (2014). A conceptual and methodological critique of internet addiction research: towards a model of compensatory internet use. Comput Hum Behav.

[14] Li D, Liau A, Khoo A (2011). Examining the influence of actual-ideal self-discrepancies, depression, and escapism, on pathological gaming among massively multiplayer online adolescent gamers. Cyberpsychology Behav Social Netw.

[15] Chen C, Zhang KZK, Zhao SJ (2015). Examining the effects of Perceived enjoyment and habit on Smartphone Addiction: the role of user type.

[16] Li W (2022). Design factors to improve the consistency and sustainable user experience of responsive Interface Design. Sustainability.

[17] Esawe AT (2022). Understanding mobile e-wallet consumers’ intentions and user behavior. Span J Mark - ESIC.

[18] Seo D, Ray S (2019). Habit and addiction in the use of social networking sites: their nature, antecedents, and consequences. Comput Hum Behav.

[19] Barberis N (2022). Problematic behaviours and flow experiences during screen-based activities as opposite outcomes of the dual process of passion and basic needs. Behav Inform Technol.

[20] Partington S, Partington E, Olivier S (2009). The dark side of flow: a qualitative study of dependence in big wave surfing. Sport Psychol.

[21] Dai C, Tai Z, Ni S. Smartphone use and psychological well-being among college students in China: a qualitative assessment. Front Psychol. 2021;12:708970. 10.3389/fpsyg.2021.708970 PMC8458628 34566786

[22] LaRose R, Eastin MS (2004). A social cognitive theory of internet uses and gratifications: toward a new model of media attendance. J Broadcast Electron Media.

[23] Oulasvirta A (2012). Habits make smartphone use more pervasive. Personal Uniquit Comput.

[24] Van Deursen AJ (2015). Modeling habitual and addictive smartphone behavior: the role of smartphone usage types, emotional intelligence, social stress, self-regulation, age, and gender. Comput Hum Behav.

[25] Hao L (2020). Avatar identification mediates the relationship between peer phubbing and mobile game addiction. Social Behav Personality: Int J.

[26] Wang J-L, Sheng J-R, Wang H-Z. The association between mobile game addiction and depression, social anxiety, and loneliness. Front Public Health. 2019:247. 10.3389/fpubh.2019.00247 PMC6743417 31552213

[27] Niedermoser DW, et al. Shopping addiction: a brief review. Practice Innovations; 2021.

[28] Yang J (2021). An empirical analysis of psychological factors based on EEG characteristics of online shopping addiction in E-Commerce. J Organizational End User Comput (JOEUC).

[29] Cha S-S, Seo B-K (2018). Smartphone use and smartphone addiction in middle school students in Korea: prevalence, social networking service, and game use. Health Psychol open.

[30] Davarpanah AS, Balaghat SR, Dadkan AR (2020). Study of the relationship between addiction and attitude to mobile messenger software and how it relates to year-old atudents in the teaching process-curriculum learning: a case study of secondary school students in khash city. J Psychologicalscience.

[31] Rajini S (2018). Study on prevalence of whatsapp addiction among medical students in a private medical college, pondicherry. Indian J Public Health Res Dev.

[32] Fang L, Liu Q (2019). Mobile SNS addiction and user continuance: an empirical investigation of wechat. Tehnički Vjesn.

[33] Gong M et al. *Understanding the role of individual differences in mobile SNS addiction* Kybernetes, 2020.

[34] Wang M-M, Wang J-J, Zhang W-N (2019). How to enhance solvers’ continuance intention in crowdsourcing contest. Online Inf Rev.

[35] Xue Y, et al. Investigating the impact of mobile SNS addiction on individual’s self-rated health. Internet Res. 2018;28(2):278–92.

[36] Li T-T, Bae S-J, Lee S-H (2022). A study on the factors affecting users’ willingness to pay, Flow and Addiction for OTT Service: focusing on China’s OTT Service platform iQIYI. J Korea Convergence Soc.

[37] Chu L-C (2012). Exploring the impact of flow experience on job performance. J Global Bus Manage.

[38] Kim J-h (2019). Effects of social network services (SNS) subjective norms on SNS addiction. J Psychol Afr.

[39] Wei P-S, Lu H-P. Why do people play mobile social games? An examination of network externalities and of uses and gratifications. Internet Res. 2014;24(3):313–31.

[40] Choi S, Aizawa K (2019). Emotype: expressing emotions by changing typeface in mobile messenger texting. Multimedia Tools and Applications.

[41] Park YW, Lee AR (2019). The moderating role of communication contexts: how do media synchronicity and behavioral characteristics of mobile messenger applications affect social intimacy and fatigue?. Comput Hum Behav.

[42] de Vries DA (2018). Social comparison as the thief of joy: emotional consequences of viewing strangers’ Instagram posts. Media Psychol.

[43] Shen J (2012). Social comparison, social presence, and enjoyment in the acceptance of social shopping websites. J Electron Commer Res.

[44] Demetrovics Z, Griffiths MD (2012). Behavioral addictions: past, present and future. J Behav Addictions.

[45] Kuss DJ, Griffiths MD (2017). Social networking sites and addiction: ten lessons learned. Int J Environ Res Public Health.

[46] Csikszentmihalyi M. Flow: the psychology of optimal experience. New York: Harper & Row; 1990.

[47] Chou T-J, Ting C-C. The role of flow experience in cyber-game addiction. CyberPsychol Behav. 2003;6(6):663–75. 10.1089/109493103322725469 14756934

[48] Hamari J (2016). Challenging games help students learn: an empirical study on engagement, flow and immersion in game-based learning. Comput Hum Behav.

[49] Fuster H, et al. Relationship between passion and motivation for gaming in players of massively multiplayer online role-playing games. Cyberpsychol Behav Soc Netw. 2014;17(5):292–297. 10.1089/cyber.2013.0349 24611801

[50] Mehmet K (2020). High schoolers’ usage intensity of mobile social media and nomophobia: investigating the mediating role of flow experience. Participatory Educational Research.

[51] Salehan M, Negahban A (2013). Social networking on smartphones: when mobile phones become addictive. Comput Hum Behav.

[52] Sun Y et al. *Understanding the Antecedents of Mobile Game Addiction: The Roles of Perceived Visibility, Perceived Enjoyment and Flow*. in *PACIS*. 2015.

[53] Trevino LK, Webster J (1992). Flow in computer-mediated communication: electronic mail and voice mail evaluation and impacts. Communication Res.

[54] Wan C-S, Chiou W-B. Psychological motives and online games addiction: Atest of flow theory and humanistic needs theory for Taiwanese adolescents. CyberPsychol Behav. 2006;9(3):317–24. 10.1089/cpb.2006.9.317 16780399

[55] Faiola A (2013). Correlating the effects of flow and telepresence in virtual worlds: enhancing our understanding of user behavior in game-based learning. Comput Hum Behav.

[56] Wood W, Neal DT (2007). A New Look at habits and the habit-goal interface. Psychol Rev.

[57] LaRose R, Lin CA, Eastin MS (2003). Unregulated internet usage: addiction, habit, or deficient self-regulation?. Media Psychol.

[58] Yang S, Wang B, Lu Y (2016). Exploring the dual outcomes of mobile social networking service enjoyment: the roles of social self-efficacy and habit. Comput Hum Behav.

[59] Tiffany ST (1990). A cognitive model of drug urges and drug-use behavior: role of automatic and nonautomatic processes. Psychol Rev.

[60] Davis FD, Bagozzi RP, Warshaw PR (1992). Extrinsic and intrinsic motivation to use computers in the workplace. J Appl Soc Psychol.

[61] Csikszentmihalyi M. Flow. The psychology of optimal experience. New York (HarperPerennial). 1990.

[62] Guo RX, Dobson T, Petrina S (2008). Digital natives, digital immigrants: an analysis of age and ICT competency in teacher education. J Educational Comput Res.

[63] Matute-Vallejo J, Melero-Polo I. Understanding online business simulation games: the role of flow experience, perceived enjoyment and personal innovativeness. Australas J Educ Technol. 2019;35(3):71–85.

[64] Sun H, Zhang P (2006). The role of moderating factors in user technology acceptance. Int J Hum Comput Stud.

[65] Chen C-C, Hsiao K-L, Li W-C (2020). Exploring the determinants of usage continuance willingness for location-based apps: a case study of bicycle-based exercise apps. J Retailing Consumer Serv.

[66] Ellis DA (2019). Do smartphone usage scales predict behavior?. Int J Hum Comput Stud.

[67] Ryan RM, Rigby CS, Przybylski A (2006). The motivational pull of video games: a self-determination theory approach. Motivation and Emotion.

[68] Przybylski AK (2012). The ideal self at play: the appeal of video games that let you be all you can be. Psychol Sci.

[69] Chatterjee S. Impact on addiction of online platforms on quality of life. Australas J Inform Syst. 2021;25:1–25.

[70] Rogers E. *Diffusion of Innovations, 3rd editio ed*. 1983, New York.

[71] Lenhart A. Teens, social media & technology overview. 2015.

[72] Bian M, Leung L (2015). Linking loneliness, shyness, smartphone addiction symptoms, and patterns of smartphone use to social capital. Social Sci Comput Rev.

[73] Tajfel H, Turner J (1986). The social identity theory of inter group behavior in S Worchel & WG Austin, editors psychology of intergroup relations.

[74] Walther JB (1996). Computer-mediated communication: Impersonal, interpersonal, and hyperpersonal interaction. Communication Res.

[75] Biocca F, Harms C, Burgoon JK (2003). Toward a more robust theory and measure of social presence: review and suggested criteria.

[76] Jennett C (2008). Measuring and defining the experience of immersion in games. Int J Hum Comput Stud.

[77] Rauwers F, Voorveld HAM, Neijens PC (2020). Explaining Perceived Interactivity effects on attitudinal responses. J Media Psychol.

[78] Tonietto GN, Barasch A (2021). Generating Content increases enjoyment by Immersing consumers and accelerating Perceived Time. J Mark.

[79] Cormier C, Langlois S. Enjoyment and self-efficacy in oral scientific communication are positively correlated to postsecondary students’ oral performance skills. Educ Sci. 2022;12(7):466.

[80] Deci EL, Ryan RM (2000). The what and why of goal pursuits: human needs and the self-determination of behavior. Psychol Inq.

[81] Islam AKMN (2022). Adverse consequences of emotional support seeking through social network sites in coping with stress from a global pandemic. Int J Inf Manag.

[82] Alexander R (2021). The neuroscience of positive emotions and affect: implications for cultivating happiness and wellbeing. Neurosci Biobehav Rev.

[83] Fredrickson BL (2001). The role of positive emotions in positive psychology: the broaden-and-build theory of positive emotions. Am Psychol.

[84] van der Linden D, Tops M, Bakker AB. The neuroscience of the flow state: involvement of the locus coeruleus norepinephrine system. Front Psychol. 2021;12:645498. 10.3389/fpsyg.2021.645498 PMC8079660 33935902

[85] Peifer C (2014). The relation of flow-experience and physiological arousal under stress—can u shape it?. J Exp Soc Psychol.

[86] Gardner B, Rebar AL. *Habit formation and behavior change*, in *Oxford research encyclopedia of psychology*. 2019.

[87] Cohn MA, Fredrickson BL (2010). In search of durable positive psychology interventions: predictors and consequences of long-term positive behavior change. J Posit Psychol.

[88] Harvey AG (2022). Applying the science of habit formation to evidence-based psychological treatments for Mental Illness. Perspect Psychol Sci.

[89] Quinn JM (2010). Can’t control yourself? Monitor those bad habits. Pers Soc Psychol Bull.

[90] Mehmood S, Taswir T (2013). The effects of social networking sites on the academic performance of students in college of applied sciences, Nizwa, Oman. Int J Arts Commer.

[91] Sadia A (2016). The relationship between Organizational Communication and Employees Productivity with New dimensions of Effective Communication Flow. J Bus Social Rev Emerg Economies.

[92] Meeks BS, Hendrick SS, Hendrick C (1998). Communication, love and relationship satisfaction. J Social Personal Relationships.

[93] Huang MH (2006). Flow, enduring, and situational involvement in the web environment: a tripartite second-order examination. Psychol Mark.

[94] Kiili K (2014). Flow framework for analyzing the quality of educational games. Entertainment Comput.

[95] Jo H, Nam D-W, Kim S-H (2011). A study on the intention of continuous use of smart phone. e-business Stud.

[96] Dwyer RJ, Kushlev K, Dunn EW (2018). Smartphone use undermines enjoyment of face-to-face social interactions. J Exp Soc Psychol.

[97] Watson D, Clark LA, Tellegen A (1988). Development and validation of brief measures of positive and negative affect: the PANAS scales. J Personal Soc Psychol.

[98] Bagozzi RP, Gopinath M, Nyer PU (1999). The role of emotions in marketing. J Acad Mark Sci.

[99] Gardner B (2015). A review and analysis of the use of ‘habit’in understanding, predicting and influencing health-related behaviour. Health Psychol Rev.

[100] Verplanken B, Aarts H (1999). Habit, attitude, and planned behaviour: is habit an empty construct or an interesting case of goal-directed automaticity?. Eur Rev Social Psychol.

[101] Klasen M (2012). Neural contributions to flow experience during video game playing. Soc Cognit Affect Neurosci.

[102] Mitas O (2012). Taking a peak at leisure travelers’ positive emotions. Leisure Sci.

[103] Turel O, Serenko A, Giles P. Integrating technology addiction and use: an empirical investigation of online auction users. MIS Q. 2011;35(4):1043–61.

[104] Singh S (2021). Assessing determinants influencing continued use of live streaming services: an extended perceived value theory of streaming addiction. Expert Syst Appl.

[105] Sunday OJ, Adesope OO, Maarhuis PL (2021). The effects of smartphone addiction on learning: a meta-analysis. Computers in Human Behavior Reports.

[106] Kwon HE (2016). Excessive dependence on mobile social apps: a rational addiction perspective. Inform Syst Res.

[107] Lopez-Fernandez O (2017). Self-reported dependence on mobile phones in young adults: a European cross-cultural empirical survey. J Behav Addictions.

[108] Osatuyi B, Turel O (2018). Tug of War between social self-regulation and habit: explaining the experience of momentary social media addiction symptoms. Comput Hum Behav.

[109] Abrahim S (2019). Structural equation modeling and confirmatory factor analysis of social media use and education. Int J Educational Technol High Educ.

[110] Jo H. Key factors influencing loyalty and satisfaction toward ERP: mediating role of Flow. J Knowl Econ. 2022;14:2138–55.

[111] Karadağ E (2015). Determinants of phubbing, which is the sum of many virtual addictions: a structural equation model. J Behav Addictions.

[112] Hair J (2017). An updated and expanded assessment of PLS-SEM in information systems research. Industrial Manage Data Syst.

[113] Munerah S, Koay KY, Thambiah S (2021). Factors influencing non-green consumers’ purchase intention: a partial least squares structural equation modelling (PLS-SEM) approach. J Clean Prod.

[114] Statista. Number of social media users worldwide from 2017 to 2027 (in billions). 2023 [cited 2023 May 17]. Available from: https://www.statista.com/statistics/278414/number-of-worldwide-social-network-users/.

[115] Ringle CM, Wende S, Becker J-M. Smartpls 3. Hamburg: SmartPLS. 2014. https://www.smartpls.com. Accessed 4 Aug 2021.

[116] Falk RF, Miller NB (1992). A primer soft modeling.

[117] Kim D, Kim B (2018). An integrative view of emotion and the dedication-constraint model in the case of coffee chain retailers. Sustainability.

[118] Chin WW, Marcolin BL, Newsted PR (2003). A partial least squares latent variable modeling approach for measuring interaction effects: results from a Monte Carlo simulation study and an electronic-mail emotion/adoption study. Inform Syst Res.

[119] Churchill GA (1979). A paradigm for developing better measures of marketing constructs. J Mark Res.

[120] Fornell C, Larcker DF (1981). Evaluating structural equation models with unobservable variables and measurement error. J Mark Res.

[121] Bagozzi RP, Yi Y, Phillips LW (1991). Assessing construct validity in organizational research. Adm Sci Q.

[122] Kiili K et al. *Flow experience as a quality measure in evaluating physically activating serious games*. in *Games and Learning Alliance: Second International Conference, GALA 2013, Paris, France, October 23–25*, 2013, Revised Selected Papers 2. 2014. Springer.

[123] McLean G, Al-Nabhani K, Marriott H (2022). Regrettable‐escapism’the negative effects of mobile app use: a retail perspective. Psychol Mark.

[124] Hoffner CA, Lee S. *Mobile phone use, emotion regulation, and well-being* Cyberpsychology, Behavior, and Social Networking, 2015. 18(7): p. 411–416. 10.1089/cyber.2014.0487 26167841

[125] Smith BW (2008). The brief resilience scale: assessing the ability to bounce back. Int J Behav Med.

[126] Vohs KD, Baumeister RF. Understanding self-regulation. Handbook Self-regul. 2004.

[127] Vallerand RJ (2003). Les passions de L’ame: on obsessive and harmonious passion. J Personal Soc Psychol.

[128] Wang C-C, Chu Y-S (2007). Harmonious passion and obsessive passion in playing online games. Social Behav Personality: Int J.

[129] Stoeber J (2011). Passion, craving, and affect in online gaming: Predicting how gamers feel when playing and when prevented from playing. Pers Indiv Differ.

[130] Kiili K (2014). Flow experience as a quality measure in evaluating physically activating collaborative serious games. Int J Serious Games.

[131] Hoffman DL, Novak TP (2009). Flow online: lessons learned and future prospects. J Interact Mark.

[132] Lin K-Y, Lu H-P (2011). Why people use social networking sites: an empirical study integrating network externalities and motivation theory. Comput Hum Behav.

[133] Kim S, Shim J, Shim J (2023). A study on the utilization of OpenAI ChatGPT as a second Language Learning Tool. J Multimedia Inform Syst.

[134] Zhou Z, Jin X-L, Fang Y (2014). Moderating role of gender in the relationships between perceived benefits and satisfaction in social virtual world continuance. Decis Support Syst.

