# Supplementary material for: Exploring the dynamics of mobile app addiction: the interplay of communication, affective factors, flow, perceived enjoyment, and habit
Source: BMC Psychol. 2023 Nov 20;11:404. doi: 10.1186/s40359-023-01440-8 (PMC10662456; doi:10.1186/s40359-023-01440-8)
Supplement: Supplementary file 1 — Supplementary Material 1 [file 40359_2023_1440_MOESM1_ESM.docx]

# Appendix A

**Table A1.** List of Model Constructs and Items

| Construct | Items | Mean | Source |
| --- | --- | --- | --- |
| Communication | CMU1 | I can get closer to people I don't see often through mobile apps. | [Abrahim et al. (2019)](#_ENREF_1) |
|  | CMU2 | I can meet various people through mobile apps. |  |
|  | CMU3 | Through mobile apps, I can have a conversation with people I meet for the first time. |  |
| Positive  Affect | POA1 | I am passionate while using mobile apps. | [Watson et al. (1988)](#_ENREF_109) |
|  | POA2 | I am proud while using mobile apps. |  |
|  | POA3 | I get inspiration while using mobile apps. |  |
| Negative  Affect | NEA1 | I suffer while using mobile apps. | [Watson et al. (1988)](#_ENREF_109) |
|  | NEA2 | I get angry while using mobile apps. |  |
|  | NEA3 | I feel annoyed while using mobile apps. |  |
| Flow | FLW1 | While using mobile apps, my attention was focused solely on them. | [Jo (2022)](#_ENREF_48) |
|  | FLW2 | I was completely focused while using mobile apps. |  |
|  | FLW3 | I was deeply immersed in mobile apps while using them. |  |
| Perceived  Enjoyment | PEN1 | It is fun to use mobile apps. | [Davis et al. (1992)](#_ENREF_22) |
|  | PEN2 | Using mobile apps is interesting. |  |
|  | PEN3 | Using mobile apps gives me pleasure. |  |
| Habit | HAB1 | I use mobile apps to kill time. | [LaRose and Eastin (2004)](#_ENREF_60) |
|  | HAB2 | I habitually use mobile apps whenever I have spare time. |  |
|  | HAB3 | I use mobile apps to relieve boredom. |  |
| Addiction | ADD1 | I was immersed in mobile apps and experienced a decrease in conversations when meeting people. | [Karadağ et al. (2015)](#_ENREF_50) |
|  | ADD2 | As I used mobile apps, the emotional and affectionate emotions of the past decreased. |  |
